# Grossesse et transplantation rénale: à propos de 10 cas

**DOI:** 10.11604/pamj.2015.20.292.4510

**Published:** 2015-03-25

**Authors:** Karima Boubaker, Madiha Mahfoudhi, Ezzeddine Abderrahim, Taieb Ben Abdallah, Adel Kheder

**Affiliations:** 1 Service de Médecine Interne A, Hôpital Charles Nicolle, Tunis, Tunisie

**Keywords:** Grossesse, transplantation rénale, corticothérapie, pregnancy, kidney transplantation, corticotherapy

## Abstract

La grossesse chez les patientes transplantées rénales est à risque de complications maternelles mais surtout fœtales. Le risque de survenue de rejet aigue ou chronique inhérent à la grossesse est faible. L'objectif de notre étude était de rapporter les grossesses survenues chez nos transplantées rénales, leurs aspects évolutifs et une revue de la littérature. L’âge moyen des patientes au moment de la transplantation rénale était de 28,5 ans. Le traitement immunosuppresseur d'entretien a associé une corticothérapie, l'azathioprine et/ou la ciclosporine A. Le délai moyen entre la transplantation rénale et la découverte de la grossesse était de 6,5 ans. L’âge moyen au moment de la conception était de 33,8 ans. Il n'ya pas eu de modifications du traitement immunosuppresseur au cours de la grossesse. La créatininémie moyenne au cours de la grossesse était stable à 104,8 µmol/l avec une créatininémie supérieure à 150 µmol/l dans 2 cas. Les complications maternelles au cours de la grossesse étaient une hypertension artérielle gravidique dans 3 cas, une protéinurie dans 3 cas, une ascension de la créatininémie au 7ème mois dans 2 cas, une cholestase hépatique gravidique dans 2 cas et une hyperuricémie dans 4 cas. Une prématurité était observée dans 3 cas en rapport avec une rupture prématurée des membranes, des contractions utérines sur utérus cicatriciel et des signes de prééclampsie dans le troisième cas. Après l'accouchement, Une hypertension artérielle était observée chez 3 patientes. On n'a pas noté de rejet aigu chez nos patientes. La créatininémie moyenne était de 195,3 µmol/l (74- 553 µmol/l). Le développement statural et psychomoteur était normal pour 9 enfants. La bonne évolution des grossesses chez les patientes transplantées rénales une planification et un suivi régulier.

## Introduction

Les patientes insuffisantes rénales chroniques ont une fertilité considérablement réduite et des grossesses à haut risque du fait des désordres hormonaux et métaboliques secondaires à l'urémie [1]. La transplantation rénale restaure la survie, la qualité de vie et la fertilité chez ces patientes [2, 3]. Approximativement 14 000 naissances dans le monde entier ont été rapportées chez les patientes transplantées d'organes [4]. La première grossesse menée à terme chez une transplantée rénale était rapportée en 1963, 3 ans après la greffe [2] et depuis des grossesses ont pu être menées à terme chez environ 2% des transplantées rénales en âge de procréer [5, 6]. La grossesse chez les patientes transplantées rénales est à risque de complications maternelles mais surtout fœtales avec des interruptions spontanées ou thérapeutiques de la grossesse, un retard de croissance intra-utérin avec prématurité et une hypotrophie fœtale. Le risque de survenue de rejet aigue ou chronique inhérent à la grossesse est faible [1, 7]. Ces grossesses doivent être planifiées avec une collaboration multidisciplinaire pour déterminer les facteurs de risque amenant à déduire le moment idéal pour la conception et adapter le traitement immunosuppressur en évitant les médicaments potentiellement tératogènes. L’évolution de la grossesse est favorable en respectant les recommandations européennes. [1]. L'objectif de notre étude était de rapporter les grossesses survenues chez nos transplantées rénales, leurs aspects évolutifs et une revue de la littérature.

## Méthodes

De 1986 à 2006, nous avons réalisé 312 transplantations rénales à partir d´un donneur vivant apparenté dans 241 cas, non apparenté dans 17 cas et à partir d´un rein de cadavre dans 54 cas. Pour les 107 femmes transplantées, on a contre-indiqué la grossesse les 2 premières années après la transplantation rénale ou en cas de traitement par le mycophénolate mofétil. Les patientes désireuses de grossesse étaient traitées par l'azathioprine d'emblée ou après ce délai en remplacement du mycophénolate mofétil. Nous avons colligé 10 grossesses survenues chez 7 patientes transplantées rénales. Toutes les grossesses étaient spontanées et non programmées.

Nous avons précisé la néphropathie initiale, l´âge au moment de la transplantation rénale, le traitement immunosuppresseur reçu, le délai entre la transplantation rénale et la conception, l’âge au moment de la conception et l’évolution de la grossesse chez chaque patiente. Les consultations en néphrologie et en gynécologie étaient mensuelles. La surveillance de la mère s'est basée sur la mesure de la pression artérielle, la pratique d'un bilan rénal, hépatique, d'hémostase et le dosage de la protéinurie de 24 heures. La surveillance fœtale s'est basée sur la perception des mouvements actifs fœtaux, la mesure du périmètre abdominal, l'auscultation des bruits de cœur fœtaux et des échographies doppler obstétricales. Après l'accouchement, on a précisé l’âge, le poids, le score d´Apgar à la naissance que le développement psychomoteur et la courbe de poids et de la taille du bébé. Un suivi des patientes transplantées après l'accouchement a comporté à chaque consultation, une mesure de la pression artérielle, la pratique de bandelettes urinaires, un bilan rénal et un dosage de la protéinurie de 24 heures.

## Résultats

L´âge moyen des patientes au moment de la transplantation rénale était de 28,5 ans (25- 35 ans). La néphropathie initiale était interstitielle dans 3 cas, glomérulaire dans 2 cas et indéterminée dans 2 cas. Une ponction biopsie rénale était pratiquée chez 4 cas. Elle a montré une glomérulonéphrite segmentaire et focale en rapport avec une maladie de Berger dans 1 cas, une glomérulonéphrite extramembraneuse classe III dans 1 cas, une néphropathie interstitielle évoquant une néphronophtise dans 1 cas et absence de glomérules dans 1 cas.

La transplantation rénale a eu lieu à partir d'un rein de donneur vivant apparenté dans 5 cas et à partir d'un rein de cadavre dans 2 cas. Le traitement immunosuppresseur d´entretien a associé une corticothérapie, l´azathioprine et/ou la ciclosporine A. Une patiente a présenté une récidive de la maladie de Berger sur le greffon rénal qui s’était manifestée cliniquement par une hématurie microscopique et une dégradation de la fonction rénale. Une hypertension artérielle était notée dans 2 cas, une protéinurie dans 2 cas, une anémie dans 1 cas et un diabète corticoinduit dans 1 cas. La créatininémie moyenne était de 103,1 mmol/l (81- 147mmol/l). Aucun cas de rejet aigu du greffon n'a été noté depuis la transplantation rénale. Les patientes avaient des cycles menstruels réguliers et n’étaient pas sous contraception. La grossesse a été découverte devant un retard des menstruations dans tous les cas. Le délai moyen entre la transplantation rénale et la découverte de la grossesse était de 6,5 ans (1-18 ans) et il était supérieur à 2 ans dans 70% des cas. L’âge moyen au moment de la conception était de 33,8 ans (29-43 ans). Il n'ya pas eu de modifications du traitement immunosuppresseur au cours de la grossesse.

La créatininémie moyenne au cours de la grossesse était stable à 104,8 µmol/l (77-188µmol/l) avec une créatininémie supérieure à 150 µmol/l dans 2 cas. Les complications maternelles au cours de la grossesse étaient une hypertension artérielle gravidique dans 3 cas, une protéinurie dans 3 cas, une ascension de la créatininémie au 7ème mois dans 2 cas respectivement de 17,6% et 21,8%, une cholestase hépatique gravidique dans 2 cas et une hyperuricémie dans 4 cas. Une prématurité était observée dans 3 cas en rapport avec une rupture prématurée des membranes, des contractions utérines sur utérus cicatriciel et des signes de prééclampsie dans le troisième cas. Le poids moyen à la naissance était de 2950 g (2100 - 3500 g) avec 4 cas d´hypotrophie fœtale (< 2800 g). Une patiente a présenté une insuffisance rénale aigue en rapport avec une infection du liquide amniotique. Les suites de couches étaient simples dans tous les cas.

Une seule patiente était perdue de vue après l'accouchement. Six patientes étaient suivies. Après un suivi moyen de 7,4 ans (2- 14 ans), la pression artérielle était normale chez 3 patientes. Une hypertension artérielle était observée chez 3 patientes. On n'a pas noté de rejet aigu chez nos patientes. La créatininémie moyenne était de 195,3 µmol/l (74- 553 µmol/l). Une ponction biopsie du greffon pratiquée chez la patiente ayant 553 µmol/l de créatininémie a montré des lésions tubulointerstitielles et vasculaires évoquant une néphropathie à la ciclosporine ainsi que des lésions de hyalinose segmentaire et focale. Cette patiente est décédée 8 ans après l'accouchement par néoplasie colique. Concernant les 3 patientes qui ont eu 2 grossesses, au bout d'un suivi moyen de 8,8 ans (4- 14 ans), une hypertension artérielle était notée dans 2 cas, une créatininémie respectivement de 112, 140 et 106 µmol/l et une protéinurie dans 1 cas. Le développement statural et psychomoteur était normal pour 9 enfants. Il était retardé pour un bébé ayant une trisomie 21dont la mère était âgée de 36 ans.

## Discussion

Nous rapportons dans cette étude rétrospective 10 grossesses survenues chez 7 patientes transplantées rénales. Les grossesses sont à faible risque puisqu'elles sont survenues au-delà de 1an de la transplantation rénale, la créatininémie était inférieure à 1 < 50 mmol/l avant la conception, une hypertension artérielle bien équilibrée sous traitement dans 2 cas et une protéinurie faible dans 2 cas. Les complications maternelles au cours de la grossesse étaient une hypertension artérielle gravidique dans 3 cas, une protéinurie dans 3 cas, une cholestase hépatique gravidique dans 2 cas et une hyper uricémie dans 4 cas. Une patiente a présenté une insuffisance rénale aigue en rapport avec une infection du liquide amniotique. Les complications fœtales étaient une prématurité dans 3 cas, une hypotrophie f'tale dans 4 cas. On a noté un cas de trisomie 21 chez un enfant dont la mère était âgée de 36 ans. Après un suivi moyen de 7,4 ans (2- 14 ans), une hypertension artérielle était observée dans 3 cas, une dysfonction chronique du greffon dans 1 cas et aucun cas de rejet aigu du greffon.

La transplantation rénale a amélioré la fertilité des patientes insuffisantes rénales et leur a donné la chance d'avoir des grossesses réussies [1]. Plusieurs registres ont étudié l’évolution des grossesses chez ces patientes et la surveillance des effets potentiels de l'exposition au traitement immunosuppresseur sur le développement fœtal et néonatal dont le « National Transplantation Pregnancy Registry » ou « NTPR » [8].

La grossesse après transplantation rénale est un évènement rare. Elle survient dans 97% des cas après la première année de transplantation rénale [9]. Toutes nos patientes ont menées une grossesse après la première année de transplantation rénale. L´âge moyen au moment de la conception est de 29±5 ans comparable avec celui de nos patientes. Il est en augmentation au fils des années [9]. Il s´agit le plus souvent de grossesses mono fœtales. Les grossesses multiples sont plus rares.

La grossesse doit être planifiée et les facteurs de risque doivent être recherchés car ils majorent de 75% les complications maternelles et/ou fœtales [6, 10, 11]. De plus la décision de chaque patiente de mener une grossesse doit être respectée. Les facteurs de risque sont un délai trop court inférieur à 2 ans entre la transplantation rénale et la grossesse, une hypertension artérielle pré-conceptionnelle même bien contrôlée par un traitement médicamenteux, une créatininémie > 150mmol/l et une protéinurie [11, 12, 13]. Le risque de complications fœtales est similaire à celle de la population générale des transplantés rénaux en particulier si la créatininémie est inférieure à 150µmol/l et la patiente est sous traitement immunosuppressur adapté. La prématurité est plus fréquente chez les transplantées rénales que dans la population générale (40% à 92% versus 12,5%) [3, 13, 15–18]. Elle dépend du délai entre le début de la grossesse et la transplantation rénale et ce risque augmente si le délai est inférieur à 1ans (51), de la présence d'une hypertension artérielle et une créatininémie > 150µmol/l [3, 11–13, 18–20]. Ainsi, le terme moyen à l´accouchement se situe à 37 semaine d'aménorrhée lorsque la créatininémie préconceptionnelle était inférieure à 121 µmol/l et en dessous de 32 SA lorsqu´elle était supérieure à 200 µmol/l [12, 20].

Vu le risque important de prématurité, une corticothérapie anténatale est indiquée dans certaines situations dans le but d´une maturation pulmonaire fœtale [21]. Dans notre série, une prématurité était observée dans 2 cas. L´hypotrophie fœtale est plus fréquente que dans la population générale (8 à 45% versus 5%) et ce indépendamment du terme de l´accouchement [18]. Le risque d'hypotrophie fœtale semble plus important lorsqu´il existe une insuffisance rénale préconceptionnelle particulièrement une créatininémie >150µmol/l, une hypertension artérielle [12, 13, 18, 22, 23] et lors d'un traitement par ciclosporine par rapport à l'azathioprine [24, 25]. Dans notre série, l'hypotrophie fœtale était observée dans 4 cas malgré une créatininémie inférieure à 150µmol/l chez nos patientes. Les infections opportunistes sur ce terrain peuvent menacer le pronostic vital et fonctionnel du fœtus [21]. Une patiente a présenté une infection du liquide amniotique. Le risque tératogène des immunosuppresseurs chez l´homme semble intervenir à fortes doses sauf pur le mycophénolate mofétil. Le mycophénolate mofétil est tératogène indépendamment de la dose pendant la période pré-conceptionnelle ou le premier trimestre [26]. Il peut être responsable de malformations crânio-faciales et cardiovasculaires fœtales en particulier des colobomes chorio-rétiniens, une atrésie du canal auditif externe avec hypoacousie, une microencéphalie, une fente orofaciale, un hypertélorisme, un micrognatisme et des anomalies de la crosse aortique ou des coronaires [26, 27]. Les corticoïdes peuvent entrainer une insuffisance surrénalienne f'tale. L´azathioprine peut engendrer une lymphopénie néonatale [24, 25].

Il est alors recommandé de planifier une grossesse si les doses des corticoïdes sont inférieures à 15mg/j, de ciclosporine inférieure à 15 mg/j et d'azathioprine inférieurs à 2 mg/kg/j [28]. Il est recommandé également d´arrêter le mycophénolate mofétil et de le remplacer par l´azathioprine 2 mois avant la conception. Dans notre série, le seul cas d'aberration chromosomique étant apparu au décours d'une grossesse tardive. Lors de l´accouchement par voie basse, le greffon rénal siégeant le plus souvent en fosse iliaque droite, ne constitue qu'exceptionnellement un obstacle praevia pour le fœtus [29]. Les naissances d´enfants vivants ont doublé ces dernières années avec un accroissement du taux de 0,19 naissances/an [9]. La mortalité néonatale est plus importante que celle de la population générale (1 à 39% versus 0,68%) [18]. Après la naissance, le développement psychomoteur des enfants est normal.

Les complications maternelles ont régressé avec comme témoin une diminution significative des interruptions thérapeutiques de grossesse [9]. Elles sont dominées par une hypertension artérielle gravidique dans 38 à 56%, de novo ou préexistante aggravée par les corticoïdes et la ciclosporine [15, 24, 25, 30]. Un traitement antihypertenseur s´impose si la pression artérielle systolique dépasse 130 mmHg et/ou la pression artérielle diastolique dépasse 80 mmHg/Hg [21]. Le traitement antihypertenseur autorisé est un bétabloquant, un alpha-méthyldopamine, l´hydralazine ou un inhibiteur calcique [31]. L´hypertension artérielle est responsable d'une pré éclampsie chez 20 à 30% des patientes qui apparaît habituellement au cours de la deuxième moitié de la grossesse [9, 10, 12, 15, 16, 20, 30] et qui nécessite une prophylaxie par le sulfate de magnésium et une délivrance dans les délais les plus brefs [21]. Dans notre étude, une hypertension artérielle gravidique était observée dans 3 cas, accompagnée d'une ascension de la créatininémie dans 1 cas, d'une protéinurie dans 2 cas et d'une hyper uricémie dans 2 cas. Nous n´avons noté aucun cas de pré éclampsie. Les perturbations du bilan hépatique au cours de la grossesse sont fréquentes et de causes multiples. Il peut s´agir d´une pré-éclampsie, d'un HELLP syndrome associant une anémie hémolytique, une augmentation des enzymes hépatiques et une thrombopénie, ou plus rarement une cholestase gravidique aigue, une cholécystite ou une hépatite virale [32, 33]. Dans notre série, une cholestase associée à une cytolyse hépatique était notée dans 1cas, une cholestase isolée dans 1 cas et une cytolyse isolée dans 1 autre cas.

Les infections particulièrement les infections urinaires, fréquentes chez les transplantées rénales étaient favorisées par l'anémie, la dénutrition secondaire à la corticothérapie et l'immunodépression [34]. Les infections opportunistes sont de gravité variable allant des mycoses vaginales à l'infection à Cytomégalovirus, la toxoplasmose et aux abcès à Aspergillus [28, 35]. Une séroconversion et une réactivation pour le Cytomégalovirus et la toxoplasmose sont possibles dans le cadre de l'immunodépression. Dans notre série, on a noté une infection amniotique dans 1 cas.

Le diabète gestationnel survient chez les transplantées rénales dans 1 à 11% des cas [9, 36]. On n'a pas observé de diabète gestationnel dans notre série. L'incidence des épisodes du rejet aigu est similaire à celle de la population générale des transplantés rénaux en particulier si la créatininémie est inférieure à 150µmol/l et la patiente est sous traitement immunosuppressur adapté [10, 14, 22, 36, 37] ou survient principalement en fin de grossesse. L'activation immunitaire aigue est inhabituelle mais peut survenir et elle est expliqué par la formation malgré l'immunosuppression, d'anticorps dirigés contre le partenaire ou le donneur [38]. De ce fait, une détection des anticorps anti-HLA doit être faite avant, pendant et après la grossesse pour ne pas passer à côté d'une activation immunitaire [38]. Nous n´avons pas observé de rejet aigu du greffon dans notre série. Le tableau clinique et biologique de rejet aigu est souvent frustre et toute suspicion doit faire pratiquer une ponction-biopsie du greffon qui confirme le diagnostic [28].

L'incidence de la dysfonction chronique du greffon est également similaire à la population générale des transplantés rénaux lorsque la créatininémie est inférieure à 150 µmol/l au moment de la conception [5, 12, 23, 36, 37]. Dans notre étude, nous a observé un seul cas de dysfonction chronique du greffon mais qui serait lié à une toxicité de la ciclosporine. La biopsie du greffon doit être pratiquée devant toute détérioration de la fonction rénale [39]. L'explication de la faible incidence de rejets aigu et chronique peut être expliquée récemment par l'expression de la protéine HLA-G normalement présente sur l'ovocyte fécondé puis sur le trophoblaste placentaire et dont le rôle est la protection du fœtus contre la réaction immunitaire par l'inhibition des lymphocytes T, des cellules NK et des cellules présentatrices d'antigènes [31]. Les grossesses chez les transplantées rénales ne semblent pas aggraver la morbi-mortalité cardio-vasculaire et la moralité globale puisque la survie de ces femmes 7 ans après l´accouchement, est de 90%, comparable à celle observée chez les femmes transplantées non enceintes [9, 36]. La grossesse augmente seulement le score vasculaire à la puerpéralité sans expression clinique [40, 41].

Le taux de césariennes est toujours beaucoup plus élevé chez les transplantées rénaux que dans la population générale, souvent supérieur à 50% [3, 16, 22, 30, 42]. Les différentes études admettent que son indication doit uniquement être obstétricale. En effet, l´accouchement par voie basse est proposé en 1ère intention chez ces patientes en dehors de toute contre-indication obstétricale. La compression des vaisseaux ou de l´uretère par la tête fœtale est sans conséquence pour le rein greffé [6].

## Conclusion

La bonne évolution des grossesses chez les patientes transplantées rénales nécessite le respect de certaines conditions. Il est important que la grossesse soit planifiée et considérée comme une grossesse à risque. Il est raisonnable d'attendre au moins une période de 1 an après la transplantation rénale pour programmer une grossesse, d'avoir une pression artérielle normale, une fonction rénale stable (créatininémie < 150 µmol/l) et l'absence de protéinurie. La survie du greffon rénal à cours et à long terme n'est pas affectée mais c'est surtout le risque de complications fœtales à type de prématurité et d'hypotrophie fœtale qui est important en cas de non respect de ces conditions. Une collaboration multidisciplinaire entre néphrologues transplanteurs, gynécologues et pédiatres est nécessaire.
